# A Meta‐Analysis of the Effects of Transcranial Direct Current Stimulation on Anger and Aggression

**DOI:** 10.1002/ab.70036

**Published:** 2025-06-06

**Authors:** Thomas F. Denson, Olivia Choy, Elizabeth Summerell, Iana Wong

**Affiliations:** ^1^ School of Psychology University of New South Wales Sydney New South Wales Australia; ^2^ School of Social Science Nanyang Technological University Singapore; ^3^ School of Psychology University of Adelaide Adelaide Australia

**Keywords:** aggression, anger, meta‐analysis, transcranial direct current stimulation

## Abstract

Anger and aggression are causes of significant suffering. Psychological methods to prevent and reduce anger and aggression have been partially successful; however, there is room for novel interventions, such as those informed by neuroscience. One such intervention is anodal transcranial direct current stimulation (tDCS), which involves administering a weak electrical current to the brain to stimulate cortical activity. In this meta‐analysis, we synthesized 93 effect sizes from 25 sham‐controlled experiments. We predicted that tDCS would reduce anger and aggression. The overall results showed no effect of tDCS on anger and aggression (Hedges' *g* = −0.03, CI_95%_ = −0.30, 0.24). Separate meta‐analyses of the dorsolateral prefrontal cortex, ventromedial prefrontal cortex, and ventrolateral prefrontal cortex showed no effects of tDCS. The meta‐analysis was limited by low power in the source articles (average power = 0.33); No study reached the sufficient sample size to detect a medium effect. Thus, there is room for more well‐powered research on the topic to determine whether tDCS may reduce aggression.

Anger is an emotion that involves a set of feelings, cognitions, and physiological responses that is often accompanied by the desire to hurt another organism (Alia‐Klein et al. 2020; Denson and Fabiansson Tan 2023). Anger is typically aroused by provocation, which often leads to aggression. Aggression is behavior intended to hurt another person (Anderson and Bushman 2002). Reactive aggression occurs when anger accompanies aggression and the goal is to harm the provocateur. By contrast, proactive aggression typically occurs in the absence of strong emotion and the intention is to obtain a goal other than harm (e.g., money, status) (Miller and Lynam 2006). Dysregulated anger and aggression are difficult to treat; thus, new interventions are needed (Lee and DiGiuseppe 2018). The aim of this meta‐analysis was to gauge the effectiveness of transcranial direct current stimulation (tDCS) in reducing anger and aggression.

Scientists have recently begun trialing tDCS as a means of attenuating anger and aggression. There have been several qualitative reviews supportive of the notion that tDCS can reduce aggression (Casula et al. 2023; Denson 2021; Denson and Choy 2025; Knehans et al. 2022; Sergiou et al. 2020; Volpe et al. 2022). Only two meta‐analyses have examined the effect of tDCS on aggression. One meta‐analysis of six effect sizes found a small, but nonsignificant aggression‐reducing effect, *d* = −0.18 (Bell and DeWall 2018), while a more recent meta‐analysis of 10 studies similarly reported a nonsignificant mean effect size (Ling et al. 2020). However, they could not examine subregions of the prefrontal cortex and other putative moderators. There have been many studies conducted on this topic since then, which we included in the present meta‐analysis.

In comparison to many psychological therapies, tDCS is appealing because recipients do not require extensive cognitive skills; tDCS is faster than a therapy session (typically 20 min per session); and it can be used at home with appropriate supervision (Charvet et al. 2020). Anodal tDCS, which is the subject of this meta‐analysis, occurs when a small electrical current is directed toward a specific region of the cortex. Anodal stimulation makes it more likely that cortical neurons will fire by regulating their excitability (Jamil and Nitsche 2017). In a typical tDCS aggression experiment, a brain region is selected, typically in the prefrontal cortex, and participants receive sham or actual stimulation. Researchers and participants are usually blind to condition. Anger is typically provided via self‐report and aggressive behavior is measured with well‐validated aggression paradigms such as the Taylor (1967) aggression paradigm or the point subtraction aggression paradigm (Cherek 1981).

The most common targets of anodal tDCS for reducing anger and aggression are the ventromedial prefrontal cortex (VMPFC), the ventrolateral prefrontal cortex (VLPFC), and the dorsolateral prefrontal cortex (DLPFC). These regions were carefully selected by researchers because of their roles in planning, inhibition, self‐control, and emotion regulation. Angry and violent people show deficits in these abilities as well as structural and functional abnormalities (Ogilvie et al. 2011; Yang and Raine 2009). The hope is that if tDCS is capable of boosting activity in these regions, it may be able to reduce anger and aggression. However, it should be noted that the prior research on this topic is limited by small sample sizes and hence, limited abilities to detect small or medium effects. Keeping this in mind, we review the underpowered literature on the effects of prefrontal tDCS on anger and aggression.

## 1 VMPFC

There is reason to suspect that tDCS over the VMPFC may influence anger and aggression. Aggressive people show structural and functional abnormalities in the VMPFC (Chester et al. 2017, 2019; Naaijen et al. 2020). Interestingly, tDCS to the VMPFC may help reduce or increase reactive aggression. Blair (2016, 2024) neurobiological model of impulsive aggression posits that the amygdala‐hypothalamic‐periaqueductal gray circuit mediates the effect of provocation on aggression. The VMPFC modulates this circuit and represents the costs and rewards of future actions (e.g., aggressive retaliation or standing down). The typical view is that the VMPFC inhibits aggressive behavior. For instance, when rewards for nonaggression (e.g., walking away unhurt) as represented in the VMPFC outweigh the rewards for aggression (e.g., getting revenge). However, the reward representation supported by the VMPFC can also increase aggression. If the perceived rewards for aggression (e.g., getting revenge) outweigh the costs (e.g., possibly getting hurt), as represented in the VMPFC, aggression may occur. In laboratory settings, aggression‐dampening norms are likely at play; thus, tDCS to the VMPFC should reduce aggression in these experiments, although it remains possible that tDCS to the VMPFC could increase aggression.

The VMPFC is also linked to emotion regulation abilities. The VMPFC may be helpful in reducing reactive aggression, in particular, due to its role in emotion regulation. A study that combined tDCS with functional magnetic resonance imaging (fMRI) showed that tDCS to the VMPFC relative to sham increased activation in the VMPFC, reduced provoked anger, and lowered aggression in a laboratory paradigm (Gilam et al. 2018). In another experiment, participants received 5 days of high‐definition anodal tDCS to the VMPFC or sham (Sergiou et al. 2022). Relative to sham, when provoked, participants who received tDCS to the VMPFC showed lower aggressive behavior and lower self‐reported aggression at the end of the 5 days. Such findings are consistent with Sergiou et al.'s (2020) review highlighting that the VMPFC should be targeted by tDCS for reducing aggression.

## 2 VLPFC

The right VLPFC is implicated in emotion regulation and inhibition and is activated during aggression paradigms (Fanning et al. 2017). It is thought to reduce aggression by lowering negative emotions and enhancing inhibitory control (e.g., Ochsner et al. 2012). For instance, a meta‐analysis of fMRI studies found that VLPFC activity was strong among aggressive people, presumably because the VLPFC was recruited to help them remain nonaggressive in the scanner (Nikolic et al. 2022; Zhang et al. 2017). Indeed, a meta‐analysis observed that the right VLPFC is recruited during inhibition tasks (Gavazzi et al. 2023) and another meta‐analysis observed strong overlap in the VLPFC during state anger and response inhibition tasks (Puiu et al. 2020).

However, one experiment found no effects of tDCS to the bilateral VLPFC on aggression, suggesting that the effects of tDCS to the VLPFC may be limited to the right hemisphere (Dambacher et al. 2015b). In support of this notion, another experiment manipulated tDCS to the right hemisphere and found that the stimulation reduced aggression following a hurtful social exclusion manipulation (Riva et al. 2015). The present meta‐analysis will investigate whether the role of the inferior frontal gyrus (IFG) in reducing anger and aggression is restricted to the right hemisphere.

## 3 DLPFC

The DLPFC is heavily involved in self‐regulatory processes including many executive functions and attention. The DLPFC also plays a role in what Blair (2024) refers to as attention‐based emotion regulation. That is, emotion regulation strategies such as cognitive reappraisal and distraction rely on the DLPFC to be effective. For instance, a recent meta‐analysis showed that the left DLPFC was reliably activated during reinterpretation and distraction (Liu et al. 2022). Thus, boosting activity to the left DLPFC via tDCS may reduce anger and aggression.

The notion that boosting activity in the left DLPFC can reduce aggression may seem at odds with the frontal asymmetry perspective on anger and aggression. This perspective states that relatively greater left prefrontal activity is associated with greater anger, aggression, and approach motivation (Harmon‐Jones 2007; Harmon‐Jones and Sigelman 2001; Peterson et al. 2008). Thus, it remains to be seen whether tDCS to the left DLPFC will increase or decrease aggression (or have no effect). For instance, one experiment with methamphetamine‐addicted participants stimulated the left DLPFC. They found that the stimulation reduced aggression on a laboratory paradigm (He et al. 2023). Another study found that stimulation to the right DLPFC increased angry rumination (Kelley et al. 2013). Thus, there is a need for a quantitative synthesis to help make sense of these mixed findings.

## 4 Additional Moderators

In addition to PFC regions and hemisphere, there are a number of unknown factors, which may influence the efficacy of tDCS on anger and aggression. The presence or absence of provocation may importantly influence how the brain works to regulate anger and aggression. Presumably, greater PFC activation is needed to help regulate emotions and inhibit aggression when participants are provoked. Other moderators are the relatively large number of parameters that require decisions by researchers (e.g., placement of the cathode, area of the electrodes, current, duration of the stimulation, and the number of sessions). We also examined sample characteristics as potential moderators (e.g., proportion of women, age, type of sample [students, clinical, forensic, etc.], and region of the world where the research was conducted).

## 5 The Present Meta‐Analysis

In the present meta‐analysis, we analyzed 93 effect sizes from 25 articles to determine whether anodal tDCS can influence anger and aggression. We expected that stimulation to the VMPFC, VLPFC, and DLPFC would reduce anger and aggression relative to sham.

## 6 Methods

### 6.1 Registration of the Meta‐Analysis, Data Availability, and Identification of Studies

The meta‐analysis was preregistered on PROSPERO (CRD42024555247; https://www.crd.york.ac.uk/PROSPERO/view/CRD42024555247). Data and R code are available on the Open Science Framework: https://osf.io/ty5sn/. We followed the Preferred Reporting Items for Systematic Reviews and Meta‐analyses (PRISMA) 2020 Guidelines (Page et al. 2021). In June 2024, we searched PsycINFO, EMBASE, PubMed, Scopus, and Proquest Dissertations and Theses. A librarian at the University of New South Wales reviewed and helped refine our search terms. Appendix A displays the search terms for each database. For example, to search PsycINFO, we used the following search strategy:

(transcranial direct current stimulation OR nibs OR tDCS OR non‐invasive brain stimulation OR noninvasive brain stimulation). ab, sh, ti

(exp Transcranial Direct Current Stimulation/)

((exp Noninvasive Brain Stimulation/OR Noninvasive Brain Stimulation. mp) OR (exp Transcranial Direct Current Stimulation/OR Transcranial Direct Current Stimulation. mp)) OR

(noninvasive brain stimulation/OR exp transcranial direct current stimulation/)

(Anger OR Angry OR Aggress* OR Violen* OR Anti‐social* OR Antisocial OR Hostil* OR bully* OR cyberbully* OR cyberaggress* OR IPV or intimate partner viol* or domestic viol*). ab, sh, ti

(exp Anger/or exp Aggressive Behavior/or exp Violence/or Aggressiveness/or exp Bullying/or exp Antisocial Behavior/or exp Criminal Behavior/)

((exp Aggressive Behavio?r/OR Aggressive Behavio?r. mp)).

We also received email alerts for databases until May 2025. We also examined the reference lists of review papers for potential articles to include. We searched Proquest to find unpublished studies and emailed the International Society for Research on Aggression for unpublished and recently published literature. All articles except one were published in peer‐reviewed articles at the time of analysis. We also contacted authors for missing data when necessary.

### 6.2 Inclusion‐Exclusion Criteria

Covidence software was used for the screening process. To be included, each study must have had an experimental manipulation consisting of an anodal tDCS condition and a sham control. Data were required to be quantitative and the participants must have been adult humans. There were no language restrictions, but the searches returned only English language research. Case studies were excluded. For the outcomes, each study must have included a measure of aggression, anger, hostility, and/or angry rumination. Trait, state, self‐report, and laboratory aggression paradigms were included. We included all of the studies that were in the two previous meta‐analyses (Bell and DeWall 2018; Ling et al. 2020), except Gilam et al. (2018), which was a within‐subjects study. Thus, we included an additional 16 experiments in the meta‐analytic database beyond these previous meta‐analyses.

### 6.3 Data Extraction

Two coders extracted all of the study characteristics and data needed to calculate effect sizes. We used Hedges' (1981) *g* as it is a measure of the standardized mean difference that is corrected for small sample size bias, thus making it more accurate for smaller samples. Most of the research in the current meta‐analyses had small samples. In most cases, *g* was derived from the means, standard deviations, and cell sizes. However, when sufficient data were unavailable to calculate *g*, we received the data from the source article authors (*n* = 8), estimated the means and standard deviations from a figure (*n* = 2), or located it in the supplementary materials (*n* = 1). We also extracted several variables, which we considered might moderate the effect sizes. For the tDCS variables, we coded the location of the anode according to the 10–20 system (e.g., F3, FpZ), hemisphere of the anode (i.e., left, right, bilateral), location of the cathode (e.g., contralateral, supraorbital, occipital, cheek), the electrode areas (cm^2^), intensity (mA), duration of stimulation (minutes), and the number of sessions. Additional moderators were world region (i.e., Asia, Europe/USA/Australia, Middle East, South America) to evaluate effect sizes in regions that differ in norms regarding displays of anger and aggression. We expected the largest effect sizes in Europe/USA/Australia. We also assessed whether participants were provoked or not and the type of outcome (e.g., reactive aggression, proactive aggression, trait, state anger). We expected that studies with provocation and those that measured reactive aggression would show larger effects sizes. We also examined sample characteristics (i.e., age, percentage of women), and sample type (e.g., clinical, forensic, healthy, students). We expected that experiments with younger, predominantly male, and forensic samples would show the largest effect sizes. Deviating from our preregistration, due to limited variability, we were unable to examine self‐report versus behavioral outcomes as anger was always self‐reported and aggression was more often observed behavior. The pre‐discussion reliability for the title and abstract screening was good, *Κ* = 0.78, as was the pre‐discussion reliability for the full text review, *Κ* = 0.63. All inconsistencies were resolved between the two coders via discussion.

Study quality was assessed by two coders with Kmet et al.'s (2004) checklist for assessing the quality of quantitative studies instrument. This instrument evaluates 14 potentially problematic areas such as sufficient blinding, appropriateness of the sample size, and the extent to which the groups are adequately described. All inconsistencies were resolved between the two coders via discussion.

### 6.4 Statistical Analyses

Because most articles had more than one effect size, we utilized multilevel meta‐analytic methods. Specifically, we used the *metafor* package in R (R Core Team 2024; Viechtbauer 2010), with study specified as a random factor. Moderators were entered into a meta‐regression in the presence of a significant *Q*‐test for heterogeneity. Heterogeneity was also assessed by *I* ^*2*^. Publication bias was examined with Egger et al's (1997) test and the trim‐and‐fill funnel plot method, cumulative meta‐analysis, PET‐PEESE, a selection model, and a p‐curve analysis. We ran two random‐effects power analyses: one for a small effect (i.e., Cohen's *d* = 0.20) and one for a medium effect (i.e., Cohen's *d* = 0.50). The 25 studies included in the meta‐analysis had an average sample size of 46, which we included as parameters along with a moderate amount of heterogeneity (*I* ^*2*^ = 0.50). Power for a small effect was 0.67 and power for a medium effect was 1.00.

## 7 Results

**Figure 1:**
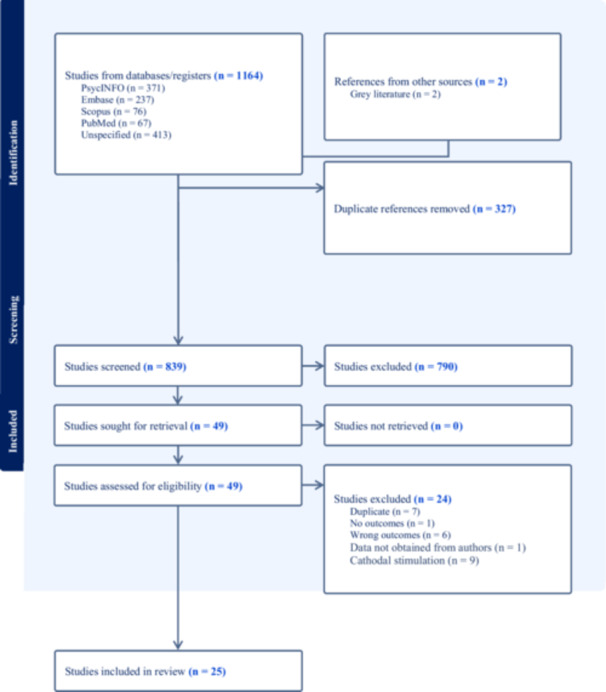
PRISMA flowchart. [Color figure can be viewed at wileyonlinelibrary.com]

The initial search produced 1,164 articles. We additionally included one unpublished experiment from our laboratory. After removing 327 duplicates, we screened 839, which resulted in 49 articles assessed for eligibility, and a total of 25 articles included in the meta‐analysis (for the PRISMA flowchart, see Figure 1). Deviating from our preregistration, we had originally intended to analyze within‐group studies (*n* = 9) and studies that used cathodal stimulation (*n *= 9) but they were too few and very diverse in terms of brain regions that were stimulated (i.e., motor cortex, cerebellum, occipital lobe, temporo‐parietal junction, VMPFC, DLPFC) to draw reasonable conclusions. A further complication is that the source articles did not report the correlations for aggressive behavior under sham and tDCS, which is required to compute standardized mean gain statistics (Becker 1988). In the PRISMA flowchart, “wrong outcomes” (*n* = 6) refers to studies that did not include a measure of anger or aggression.

**Figure 2:**
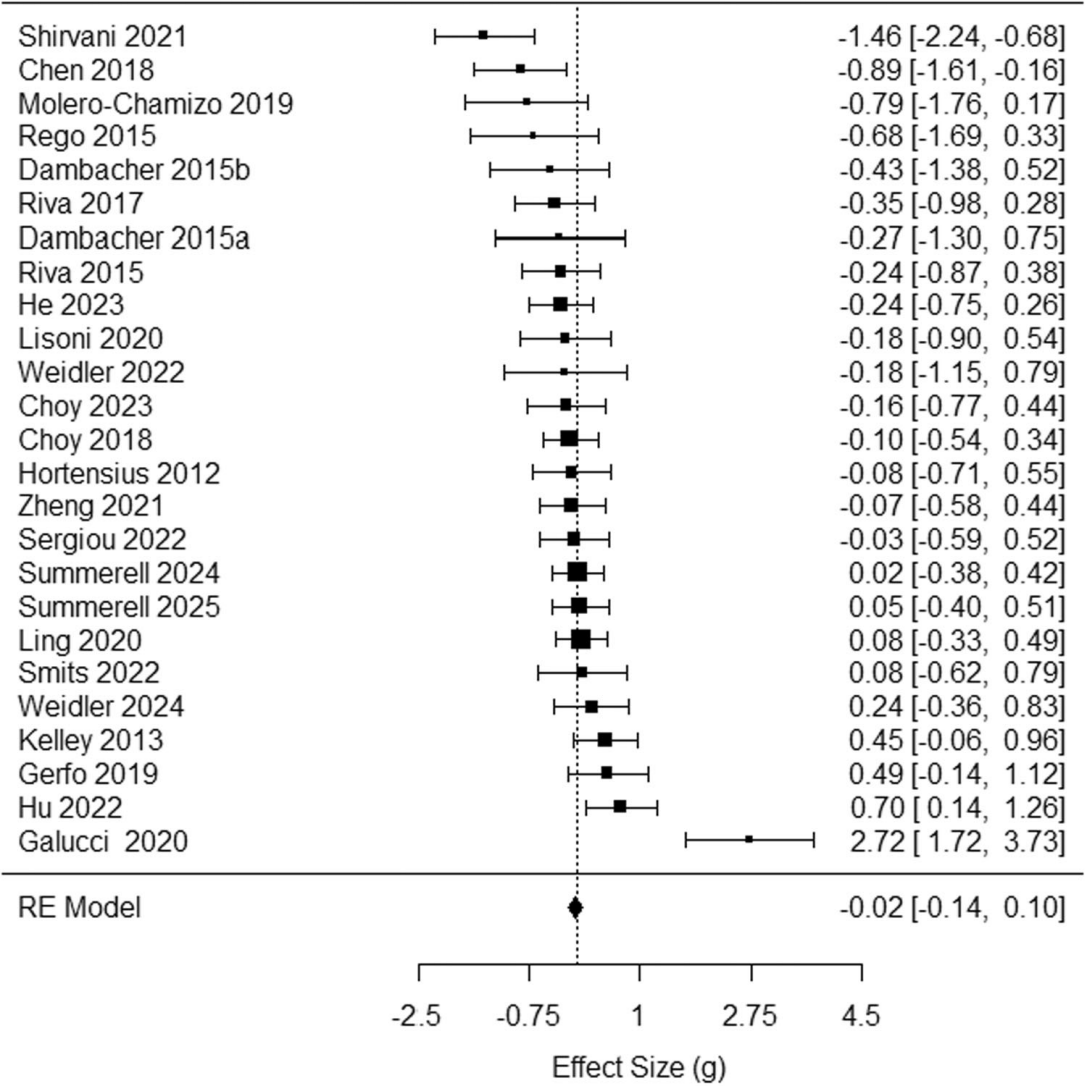
Univariate forest plot for between‐group articles using anodal stimulation to reduce anger and aggression.

For the final data set, there were 25 articles with anodal stimulation and between‐group comparisons (*k *= 93). Table 1 shows that study characteristics. Appendix B displays a list of studies included in the meta‐analysis. Figure 2 shows the forest plot for all 25 articles (one effect size per article). Each article's effect sizes were averaged. Thus, each point represents the average effect size for that article. Effect sizes ranged from −1.64 to 3.49. Some of the effects tested come from a relatively small number of effect sizes. Analyses with fewer than 10 effect sizes should be interpreted cautiously (Kepes et al. 2012).

**Table 1:** Summary of studies included in the meta‐analysis.

Study	Anode	Provocation	Outcome(s)	Current (mA)	Duration (mins)	No. of sessions	Sample type	Country
Chen 2018	rVLPFC	No	Proactive & reactive aggression	2.0	12.5	1	Healthy	Taiwan
Choy 2018	Bilateral DLPFC	No	Intentions to harm	2.0	20	1	Healthy	USA
Choy 2023	Bilateral VMPFC	Yes	Reactive aggression	2.0	20	3	Healthy	Singapore
Dambacher 2015a	rDLPFC	Yes	Proactive, reactive, & trait aggression	2.0	12.5	1	Students	Netherlands
Dambacher 2015b	lVLPFC & rVLPFC	Yes	Reactive & proactive aggression	1.5	21.75	1	Healthy	Netherlands
Gallucci et al. 2020	lVLPFC & rVLPFC	Yes	Reactive aggression	1.5	20	1	Healthy	Italy
Gerfo 2019	rTPJ & bilateral VMPFC	Yes	3rd party punishment	1.5	20	1	Students	Italy
He 2023	lDLPFC	Yes	Proactive & reactive aggression	2.0	20	10	Methamphetamine addicted	China
Hortensius 2012	lDLPFC & rDLPFC	Yes	Reactive aggression & state anger	2.0	15	1	Students	Netherlands
Hu 2022	rDLPFC	Yes	Reactive aggression & state anger	1.5	20	1	Students	China
Kelley 2013	lDLPFC & rDLPFC	Yes	Angry rumination	2.0	15	1	Students	USA
Ling 2020	Bilateral DMPFC	Yes	Intentions to harm	2.0	20	1	Students	USA
Lisoni 2020	rDLPFC	No	Trait physical aggression and trait anger	2.0	20	15	Borderline personality disorder	Italy
Molero‐Chamizo 2019	Bilateral DLPFC	No	Trait verbal aggression and trait hostility	1.5	15	3	Forensic	Spain
Rêgo 2015	lDLPFC & rDLPFC	No	State hostility	2.0	15	1	Students	Brasil
Riva 2015	rVLPFC	Yes	Reactive aggression	1.5	20	1	Students	Italy
Riva 2017	rVLPFC	Yes	Proactive & reactive aggression	1.5	20	1	Students	Italy
Sergiou 2022	Bilateral VMPFC	Yes	Reactive aggression and trait reactive‐pro active aggression	2.0	20	2	Forensic	Netherlands
Shirvani et al. (2021)	lDLPFC	No	Trait aggression	1.5	20	10	Traumatic brain injury	Iran
Smits 2022	VLPFC	No	Trait aggression	1.25	20	5	PTSD	Netherlands
Summerell 2024a	Bilateral VMPFC	Yes	Reactive aggression	1.5	13	1	Intoxicated and sober students	Australia
Summerell 2024b	rVLPFC	Yes	State anger	1.5	20	1	Intoxicated and sober students	Australia
Weidler 2022	rDLPFC	Yes	Reactive aggression	1.5	20	1	Healthy, smokers, & alcohol dependent	Germany
Weidler 2024	rDLPFC	Yes	Reactive aggression	1.5	20	1	Healthy	Germany
Zheng 2021	lDLPFC & rDLPFC	No	Proactive aggression	1.5	20	1	Students	China

Abbreviations: DLPFC = dorsolateral prefrontal cortex, DMPFC = dorsomedial prefrontal cortex, *l* = left hemisphere, PTSD = posttraumatic stress disorder, *r* = right hemisphere, TPJ = temporoparietal junction, VLPFC = ventrolateral prefrontal cortex, VMPFC = ventromedial prefrontal cortex.

The overall effect of tDCS on combined anger and aggression was not significant, *g* = −0.03 (CI_95%_ = −0.30, 0.24), *SE* = 0.14, *z* = −0.19, *p* = 0.85, *Q*(92) = 361.09, *I* ^*2*^ = 81.89. In a sensitivity analysis, we removed one outlier (i.e., Gallucci et al. 2020). The mean effect size showed a greater reduction in combined anger and aggression but was still not significant, *g* = −0.11 (CI_95%_ = −0.27, 0.05), *k* = 86, *z* = −1.37, *p* = 0.17, *Q*(86) = 191.38, *p* < 0.0001, *I* ^*2*^ = 58.15. Separate analyses of the anger and aggression effects revealed null effects for anger, *g* = 0.04 (CI_95%_ = −0.15, 0.23), *k* = 23, *z* = 0.41, *p* = 0.68, *Q*(22) = 40.25, *p* = 0.010, *I* ^*2*^ = 39.05, and aggression, *g* = −0.04 (CI_95%_ = −0.38, 0.31), *k* = 70, *z* = −0.21, *p* = 0.84, *Q*(69) = 319.41, *p* < 0.0001, *I* ^*2*^ = 83.39.

However, because we suspected that the effects of region of stimulation might be influenced by the moderators, we tested for moderation separately within the VMPFC, DLFPC, and VLPFC.

### 7.1 VMPFC

The mean effect size was not significant for studies that used anodal stimulation to manipulate activity in the VMPFC, (*k* = 13), *g* = 0.07 (CI_95%_ = −0.12, 0.26), *SE* = 0.10, *z* = 0.71, *p* = 0.48. However, there was significant heterogeneity, *Q*(12) = 36.63, *p* = 0.0003, *I* ^*2*^ = 16.04. Gender was a significant moderator, *Q*(1) = 4.44, *p* = 0.035, such that studies with more women showed that tDCS increased anger and aggression to a greater extent than studies with relatively fewer women, *estimate* = 0.01 (CI_95%_ = 0.001, 0.014), *SE* = 0.003, *z* = 2.11, *p* = 0.035.

### 7.2 VLPFC

The mean effect size was not significant for studies that used anodal stimulation to manipulate activity in the VLPFC, (*k* = 31), *g* = 0.15 (CI_95%_ = −0.67, 0.96), *SE* = 0.42, *z* = 0.35, *p* = 0.73. However, there was significant heterogeneity, *Q*(30) = 211.54, *p* < 0.0001, *I* ^*2*^ = 91.98. Although there were too few electrodes placed at F5 to conduct a significance test, one study reported that electrodes placed at F5 (*g* = 3.35, CI_95%_ = 2.71, 3.99, *k* = 3) showed descriptively larger anger and aggression compared to electrodes placed at F6 (*g* = 0.11, CI_95%_ = −0.67, 0.90, *k* = 20). However, this effect for F5 was based on one study. Gender was also a significant moderator, *Q*(1) = 12.26, *p* = 0.0005, such that studies with more women showed that tDCS decreased effect sizes relative to studies with fewer women, *estimate* = −0.01 (CI_95%_ = −0.018, −0.005), *SE* = 0.003, *z* = −3.50, *p* = 0.0005. With the outlier removed, the effect became stronger, but remained nonsignificant, *g* = −0.21 (CI_95%_ = −0.45, 0.02), *k* = 25, *z* = −1.77, *p* = 0.076, *Q*(24) = 41.94, *p* = 0.013, *I* ^*2*^ = 38.06.

### 7.3 DLPFC

The mean effect size was not significant for studies that used anodal stimulation to manipulate activity in the DLPFC, (*k* = 44), *estimate* = −0.16 (CI_95%_ = −0.43, 0.12), *SE* = 0.14, *z* = −1.12, *p* = 0.26. However, there was significant heterogeneity, *Q*(43) = 98.59, *p* < 0.0001, *I* ^*2*^ = 66.34. Anode placement was a significant moderator, *Q*(2) = 7.99, *p* = 0.018, such that electrodes placed at F3 (*g* = −0.40, CI_95%_ = −0.87, 0.07, *k* = 15) showed moderate decreases in anger and aggression relative to electrodes placed at F4 (*g* = 0.13, CI_95%_ = −0.15, 0.41, *k* = 23), *estimate* = 0.43 (CI_95%_ = 0.12, 0.73), *SE* = 0.16, *z* = 2.75, *p* = 0.006. However, both confidence intervals crossed zero. Cathode placement was also a significant moderator; however, there were too few effect sizes in each cathode group to make meaningful comparisons.

Anode size was a significant moderator, *Q*(2) = 14.60, *p* = 0.0007, such that smaller electrodes of 25 cm^2^ showed significant reductions in anger and aggression (*g* = −0.26, CI_95%_ = −0.44, −0.08, *k* = 13) whereas electrodes of 35 cm^2^ did not (*g* = 0.05, CI_95%_ = −0.20, 0.29, *k* = 29). One study using very small electrodes of 10.5 cm^2^ also showed a significant reduction in trait aggression (*g* = −1.46, CI_95%_ = −2.01, −0.91, *k* = 2) (Shirvani et al. 2021). Electrodes areas of 35 cm^2^ and 100 cm^2^ did not significantly influence the effect sizes.

The presence of provocation was also a significant moderator, *Q*(1) = 5.54, *p* = 0.019, such that studies in which participants were not provoked (*g* = −0.49, CI_95%_ = −0.91, −0.07, *k* = 14) showed significantly lower aggression relative to studies in which participants were provoked (*g* = 0.09, CI_95%_ = −0.18, 0.37, *k* = 30), *estimate* = 0.57 (CI_95%_ = 0.10, 1.05), *SE* = 0.24, *z* = 2.35, *p* = 0.019. World region was a significant moderator, *Q*(3) = 12.44, *p* = 0.006, but this result was due to one study conducted in Iran (Shirvani et al. 2021).

### 7.4 Publication Bias

**Figure 3:**
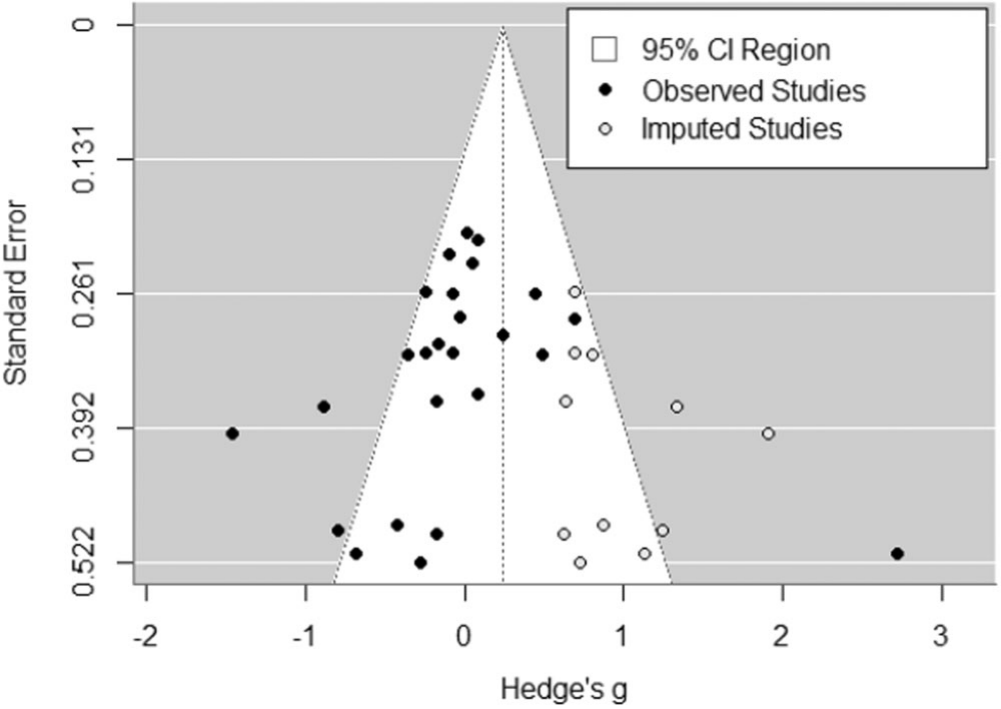
Funnel plot showing publication bias. The data are one effect size per study.

**Figure 4:**
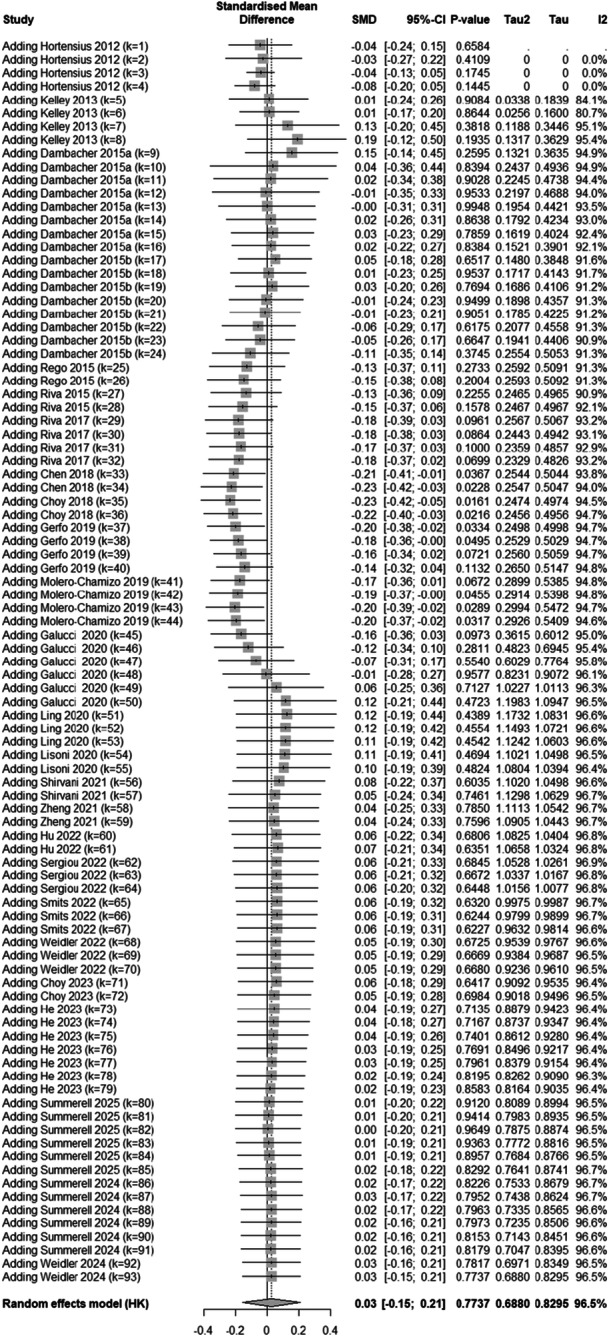
Results from the cumulative meta‐analysis.

**Figure 5:**
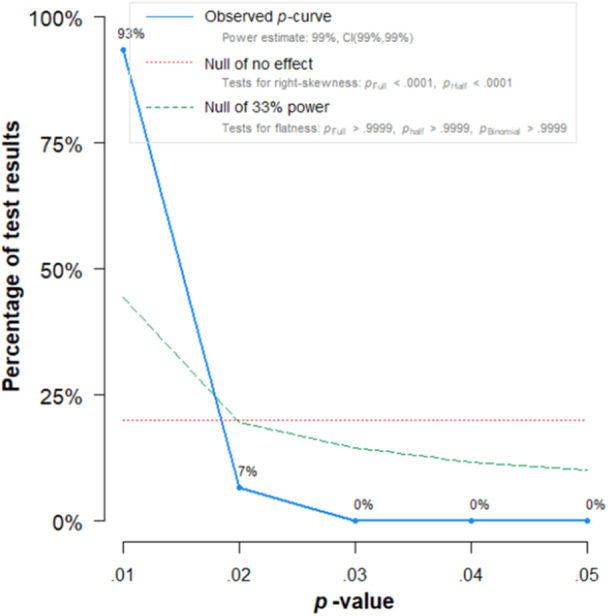
P‐curve of the random effects meta‐analysis. [Color figure can be viewed at wileyonlinelibrary.com]

There was mixed evidence of publication bias. The funnel plot on the univariate data showed evidence of publication bias. Specifically, 11 studies were suspected missing on the right side and none on the left (Figure 3). However, Egger et al's (1997) test revealed no evidence of funnel plot asymmetry, *z* = −0.42, *p* = 0.68. A random effects trim and fill analysis showed 8 missing studies, which increased the estimated mean effect size to *g* = 0.13 (CI_95_ = −0.06, 0.31), *t* = 1.41, *p* = 0.16. We conducted a cumulative meta‐analysis using the *meta* package in R (Balduzzi et al. 2019). This cumulative meta‐analysis (Figure 4) showed, with few exceptions, no deviation from zero. Effect sizes were largest when studies conducted between 2015 and 2019 were added to the meta‐analysis. A PET‐PEESE analysis revealed a nonsignificant effect size for the PET, *g* = 0.023, *SE* = 0.087, *t* = 0.26, *p* = 0.79, revealing no evidence of publication bias. Because the PET result was not‐significant, we did not fit a PEESE model. Furthermore, a selection model revealed significant publication bias, *χ* ^2^(1) = 9.99, *p* = 0.002. Finally, a p‐curve analysis of the random effects meta‐analysis showed significant right skew, suggesting that p‐hacking and other questionable research practices may not have affected the results of this meta‐analysis (Figure 5). The disclosure table for the p‐curve analysis is available here: https://osf.io/ty5sn/.

### 7.5 Quality Assessment

Table 2 displays the results of the quality assessment. The source articles were rated well on describing the study question, selecting appropriate study designs, appropriate analyses and reporting, describing the outcomes, and reporting blinding of participants. The source articles were less well rated on sample size, controlling for confounds, describing the random allocation method, and reporting blinding of investigators. In particular, no study had a sufficient sample size to detect a medium effect (i.e., *g* = 0.50).

**Table 2:** Quality Assessment.

Criterion	Yes	Partially	No
Objective sufficiently described	100%	0%	0%
Study design evident and appropriate	100%	0%	0%
Method of participant selection described and appropriate	44%	44%	12%
Participant characteristics sufficiently described	92%	8%	0%
Random allocation method was described	16%	76%	8%
Blinding of investigator was reported	60%	40%	0%
Blinding of participants was reported	92%	0%	8%
Outcome measures well defined	100%	0%	0%
Sample size was appropriate	0%	84%	16%
Analytic methods justified and appropriate	96%	4%	0%
Some estimate of variance reported	92%	0%	8%
Control for confounds	56%	12%	32%
Results reported in sufficient detail	100%	0%	0%
Conclusions supported by the results	100%	0%	0%

### 7.6 Statistical Power

**Figure 6:**
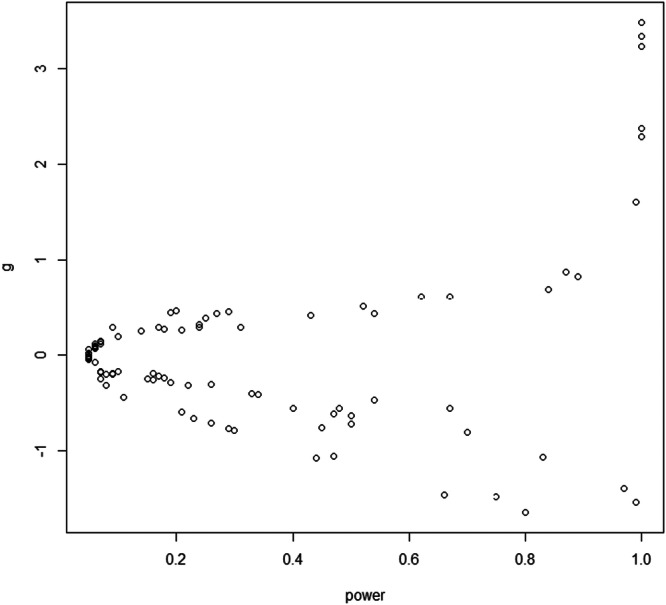
Plot showing statistical power (x‐axis) as a function of observed effect sizes (y‐axis).

During the extraction process, it became apparent that the sample sizes were typically too small to observe adequate power. Some effect sizes had less than 10 participants per group. No study reached the sufficient sample size of 64 per group to detect a medium effect (Hedge's *g* = 0.50) at 80% power. We used GPower (Faul et al. 2007) to calculate achieved power for each effect size. The average power in these experiments was 0.33. As shown in Figure 6, power was only adequate (0.80) for very large effect sizes (*g* approximately greater than |1.00 | ), which were rarely observed in the primary experiments.

## 8 Discussion

The aim of the present meta‐analysis was to quantify the effects of tDCS on anger and aggression, with a particular emphasis on the VMPFC, VLPFC, and DLPFC. Results did not support our hypothesis that stimulation to these brain regions would lessen anger and aggression. There was suggestive evidence that electrodes placed over the left DLPFC may reduce aggression relative to electrodes placed over the right DLPFC; however, it appears that the reduction of aggression was limited to studies that did not provoke participants (e.g., proactive aggression), making it irrelevant to reactive aggression. Placement of anodal electrodes 25 cm^2^ or less over the DLPFC was associated with significant reductions in anger and aggression. Thus, at best, some practical research advice might be to use small electrodes with the anode on the left DLPFC at F3 and proactive aggression paradigms. The other results failed to support an aggression‐reducing role of the VLPFC and VMPFC.

Our null effects are consistent with two prior meta‐analyses that also observed null effects of tDCS on aggression (Bell and DeWall 2018; Ling et al. 2020). Ling et al. (2020) included 10 studies and found an effect of *d* = − 0.028, which is nearly identical to the effect size we observed in this updated meta‐analysis of 25 studies. The question remains as to why we failed to observe a significant effect of tDCS on anger and aggression. One possibility is that these brain regions are not implicated in the control of anger and aggression. We find this possibility unlikely given the preponderance of evidence from neuroimaging and lesion studies (Blair 2024; Fanning et al. 2017; Peng et al. 2024; Sorella et al. 2021). Another possibility is that the tDCS parameters used in the source articles may have been insufficient to stimulate the brain to exert the desired psychological effects on anger and aggression. Similarly, because tDCS affects relatively large portions of the brain, it may not be precise enough to target the specific sub‐regions of the cortex that it needs to to affect anger and aggression. Perhaps research using repetitive transcranial magnetic stimulation (rTMS) with its improved spatial properties may be a more powerful way to test stimulation effects on anger and aggression. Yet another possibility is that the null results here represent a true null effect of tDCS on anger and aggression. That is, tDCS may have no impact on psychological functioning that could decrease or increase aggression. Anger and aggression are complicated phenomena and using tDCS may not elicit changes to the processes that underlie anger and aggression.

Another possibility for the null results is that the studies are simply low powered. Indeed, our analysis of statistical power in the source articles showed achieved power as much lower than the 0.80 that is typically desired in the social sciences (Cohen 2013). It is highly recommended that researchers maximize their sample sizes and minimize sources of error that might compromise internal validity. The quality assessment speaks to this issue as well; in particular, selecting appropriate samples and controlling for confounds may reduce error. It is worth keeping in mind that the magnitude of these effects likely to be small‐to‐moderate. That is, when making decisions about sample size and running power analyses, researchers would be advised to incorporate realistic effect size estimates. For instance, psychological treatments for anger and aggression, which last multiple sessions often show relatively small effect sizes (Hedges' *g*s = −0.21 for anger and −0.10 for aggression; Ciesinski et al. 2022). Thus, it is unlikely that a single session of tDCS may show a medium or large effect. Researchers should adjust their expectations regarding expected effect sizes.

The present meta‐analysis was limited in some ways beyond the low power inherent in the primary experiments. One is that there was an insufficient number of effect sizes to truly explore the moderators for each of the brain regions. This made it impossible to examine higher order interactions (e.g., anode location × aggression type × sample type). Indeed, some individual studies reported interactions between personal characteristics (e.g., alcohol dependence, genetic risk) and tDCS (e.g., Weidler et al. 2022, 2024). However, because studies examining higher‐order interactions were too few and varied, we could not meta‐analyze these findings. Future, high‐powered experiments which incorporate individual differences might reveal for whom tDCS can be most effective. Having said that, detecting interactions will likely require a large number of participants. Hyatt et al. (2022) conducted simulations based on average effects size for aggression studies and reported that detecting two‐way interactions may require thousands of participants. Although detecting main effects requires fewer participants, the source articles were still underpowered in this regard. Thus, perhaps large pre‐registered experiments may be more feasible than testing interactions.

With any meta‐analysis, the question of publication bias is important. We conducted multiple tests of publication bias and found mixed evidence with random and fixed effects funnel plots displaying some file drawer papers; whereas Egger et al.'s (1997) test found no such asymmetry. Thus, there may be some file drawer papers showing aggression‐increasing effects of tDCS. A cumulative meta‐analysis showed little differences in effect sizes over time and the PET analysis showed no evidence of publication bias. Finally, the p‐curve analysis revealed no evidence of p‐hacking. Taken as a whole, there appears to be little evidence of publication bias beyond a few file drawer papers.

Although diversity in this study area is welcome, the heterogeneity in experimental procedures may have contributed to the null effect. Most experiments were conducted in accordance with standards for good research practice. For example, participants were blind to sham or anodal tDCS in nearly all of the experiments. Many made use of well‐validated aggression paradigms such as the Taylor (1967) aggression paradigm or point subtraction aggression paradigm (Cherek 1981). These powerful tools have been in frequent use by aggression researchers for decades and are desirable because they can measure aggressive behavior in the heat of the moment. However, other studies used trait measures and self‐reported anger, which may be less sensitive to the effects of tDCS. Similarly, there were too few studies to examine unique effects of tDCS on anger and aggression. The majority of studies also involved only one session of anodal stimulation. However, there is evidence that larger effects may be obtained from multi‐session noninvasive brain stimulation interventions compared to single‐session ones (Song et al. 2019).

In sum, the current meta‐analysis failed to show a significant effect of tDCS on anger and aggression. However, there was suggestive evidence that stimulating the left DLPFC with small electrodes may reduce unprovoked aggression. Furthermore, more work is needed with high‐powered experimental procedures to truly test whether tDCS should be used to treat problems with anger and aggression. It is still early days as all articles included in this meta‐analysis were published from 2012 onwards. Thus, the future may yet show that tDCS works for some people in some situations.

## Conflicts of Interest

The authors declare no conflicts of interest.

## Data Availability

The data that support the findings of this study are openly available in Open Science Framework at https://osf.io/ty5sn/.

## Appendix A Search Terms in Databases

PsycINFO:

(transcranial direct current stimulation OR nibs OR tDCS OR noninvasive brain stimulation OR noninvasive brain stimulation). ab, sh, ti

(exp Transcranial Direct Current Stimulation/)

((exp Noninvasive Brain Stimulation/OR Noninvasive Brain Stimulation. mp) OR (exp Transcranial Direct Current Stimulation/OR Transcranial Direct Current Stimulation. mp)) OR

(noninvasive brain stimulation/OR exp transcranial direct current stimulation/)

(Anger OR Angry OR Aggress* OR Violen* OR Antisocial* OR Antisocial OR Hostil* OR bully* OR cyberbully* OR cyberaggress* OR IPV or intimate partner viol* or domestic viol*). ab, sh, ti

(exp Anger/or exp Aggressive Behavior/or exp Violence/or Aggressiveness/or exp Bullying/or exp Antisocial Behavior/or exp Criminal Behavior/)

((exp Aggressive Behavio?r/OR Aggressive Behavio?r. mp))

ProQuest Dissertations and Theses: (https://www.proquest.com/):

ALL((“transcranial direct current stimulation” OR tDCS OR “noninvasive brain stimulation” OR “noninvasive brain stimulation”) AND (Anger OR Angry OR Aggress* OR Violen* OR Antisocial* OR Antisocial OR Hostil* OR bully* OR cyberbully* OR cyberaggress* OR IPV or “intimate partner viol*” or “domestic viol*”))

PubMed:

(transcranial direct current stimulation[Text Word] OR “transcranial direct current stimulation”[Mesh] OR tDCS[Text Word] OR noninvasive brain stimulation[Text Word] OR nibs[Text Word] OR noninvasive brain stimulation[Text Word]) AND (Aggress*[Text Word] OR Violen*[Text Word] OR Antisocial*[Text Word] OR Antisocial[Text Word] OR Hostil*[Text Word] OR bully*[Text Word] OR cyberbully*[Text Word] OR cyberaggress*[Text Word] OR intimate partner viol*[Text Word] or domestic viol*[Text Word] OR “intimate partner violence”[Mesh] OR “Aggression”[Mesh] OR “hostility”[Mesh] OR “Violence”[Mesh])

Embase:

Same syntax as PsycINFO

(transcranial direct current stimulation OR nibs OR tDCS OR noninvasive brain stimulation OR noninvasive brain stimulation). ab, sh, ti

(exp Transcranial Direct Current Stimulation/)

((exp Noninvasive Brain Stimulation/OR Noninvasive Brain Stimulation. mp) OR (exp Transcranial Direct Current Stimulation/OR Transcranial Direct Current Stimulation. mp)) OR

(noninvasive brain stimulation/OR exp transcranial direct current stimulation/)

(Anger OR Angry OR Aggress* OR Violen* OR Antisocial* OR Antisocial OR Hostil* OR bully* OR cyberbully* OR cyberaggress* OR IPV or intimate partner viol* or domestic viol*). ab, sh, ti

(exp Anger/or exp Aggressive Behavior/or exp Violence/or Aggressiveness/or exp Bullying/or exp Antisocial Behavior/or exp Criminal Behavior/)

((exp Aggressive Behavio?r/OR Aggressive Behavio?r. mp))

Scopus:

Combine these with AND in a separate search box for ALL FIELDS.

transcranial direct current stimulation OR tDCS OR noninvasive brain stimulation OR noninvasive brain stimulation OR nibs

AND

Anger OR Angry OR aggress* OR violent OR violence OR antisocial OR antisocial OR hostil* OR bullying OR cyberbullying OR cyberaggression OR ipv OR “intimate partner violence” OR “domestic violence”

## Appendix B List of Articles included in the Meta‐analysis

Chen, C. Y. (2018). Right ventrolateral prefrontal cortex involvement in proactive and reactive aggression: A transcranial direct current stimulation study. *NeuroReport*, *29*(17), 1509‐1515. DOI: 10.1097/WNR.0000000000001144

Choy, O., Raine, A., & Hamilton, R. H. (2018). Stimulation of the prefrontal cortex reduces intentions to commit aggression: a randomized, double‐blind, placebo‐controlled, stratified, parallel‐group trial. *Journal of Neuroscience*, *38*(29), 6505‐6512. DOI:10.1523/JNEUROSCI.3317‐17.2018

Choy, O., Tan, G., & Wong, Y. C. (2023). The Effect of Multi‐Session Prefrontal Cortical Stimulation on Aggression: A Randomized, Double‐Blind, Parallel‐Group Trial. *Life*, *13*(8), 1729. 10.3390/life13081729

Dambacher, F., Schuhmann, T., Lobbestael, J., Arntz, A., Brugman, S., & Sack, A. T. (2015a). Reducing proactive aggression through noninvasive brain stimulation. *Social cognitive and affective neuroscience*, *10*(10), 1303‐1309. doi:10.1093/scan/nsv018

Dambacher, F., Schuhmann, T., Lobbestael, J., Arntz, A., Brugman, S., & Sack, A. T. (2015b). No effects of bilateral tDCS over inferior frontal gyrus on response inhibition and aggression. *PloS one*, *10*(7), e0132170. doi:10.1371/journal. pone.0132170

Gallucci, A., Riva, P., Lauro, L. J. R., & Bushman, B. J. (2020). Stimulating the ventrolateral prefrontal cortex (VLPFC) modulates frustration‐induced aggression: A tDCS experiment. *Brain stimulation*, *13*(2), 302‐309. 10.1016/j. brs.2019.10.015

He, J., Wang, R., Li, J., Jiang, X., Zhou, C., & Liu, J. (2023). Effect of transcranial direct current stimulation over the left dorsolateral prefrontal cortex on the aggressive behavior in methamphetamine addicts. *Journal of psychiatric research*, *164*, 364‐371. 10.1016/j. jpsychires.2023.06.038

Hortensius, R., Schutter, D. J., & Harmon‐Jones, E. (2012). When anger leads to aggression: Induction of relative left frontal cortical activity with transcranial direct current stimulation increases the anger–aggression relationship. *Social cognitive and affective neuroscience*, *7*(3), 342‐347. doi:10.1093/scan/nsr012

Hu, X., Zhang, Y., Liu, X., Guo, Y., Liu, C., & Mai, X. (2022). Effects of transcranial direct current stimulation over the right dorsolateral prefrontal cortex on fairness‐related decision‐making. *Social Cognitive and Affective Neuroscience*, *17*(8), 695‐702. 10.1093/scan/nsac004

Kelley, N. J., Hortensius, R., & Harmon‐Jones, E. (2013). When anger leads to rumination: Induction of relative right frontal cortical activity with transcranial direct current stimulation increases anger‐related rumination. *Psychological science*, *24*(4), 475‐481.

Ling, S., Raine, A., Choy, O., & Hamilton, R. (2020). Effects of prefrontal cortical stimulation on aggressive and antisocial behavior: A double‐blind, stratified, randomized, sham‐controlled, parallel‐group trial. *Journal of experimental criminology*, *16*, 367‐387.

Lisoni, J., Miotto, P., Barlati, S., Calza, S., Crescini, A., Deste, G.,… & Vita, A. (2020). Change in core symptoms of borderline personality disorder by tDCS: A pilot study. *Psychiatry Research*, *291*, 113261.

Gerfo, E. L., Gallucci, A., Morese, R., Vergallito, A., Ottone, S., Ponzano, F.,… & Lauro, L. J. R. (2019). The role of ventromedial prefrontal cortex and temporo‐parietal junction in third‐party punishment behavior. *Neuroimage*, *200*, 501‐510.

Molero‐Chamizo, A., Riquel, R. M., Moriana, J. A., Nitsche, M. A., & Rivera‐Urbina, G. N. (2019). Bilateral prefrontal cortex anodal tDCS effects on self‐reported aggressiveness in imprisoned violent offenders. *Neuroscience*, *397*, 31‐40.

Rêgo, G. G., Lapenta, O. M., Marques, L. M., Costa, T. L., Leite, J., Carvalho, S.,… & Boggio, P. S. (2015). Hemispheric dorsolateral prefrontal cortex lateralization in the regulation of empathy for pain. *Neuroscience letters*, *594*, 12‐16.

Riva, P., Romero Lauro, L. J., DeWall, C. N., Chester, D. S., & Bushman, B. J. (2015). Reducing aggressive responses to social exclusion using transcranial direct current stimulation. *Social cognitive and affective neuroscience*, *10*(3), 352‐356.

Riva, P., Gabbiadini, A., Romero Lauro, L. J., Andrighetto, L., Volpato, C., & Bushman, B. J. (2017). Neuromodulation can reduce aggressive behavior elicited by violent video games. *Cognitive, Affective, & Behavioral Neuroscience*, *17*, 452‐459.

Sergiou, C. S., Santarnecchi, E., Romanella, S. M., Wieser, M. J., Franken, I. H., Rassin, E. G., & van Dongen, J. D. (2022). Transcranial direct current stimulation targeting the ventromedial prefrontal cortex reduces reactive aggression and modulates electrophysiological responses in a forensic population. *Biological Psychiatry: Cognitive Neuroscience and Neuroimaging*, *7*(1), 95‐107.

Shirvani, S., Davoudi, M., Shirvani, M., Koleini, P., Hojat Panah, S., Shoshtari, F., & Omidi, A. (2021). Comparison of the effects of transcranial direct current stimulation and mindfulness‐based stress reduction on mental fatigue, quality of life and aggression in mild traumatic brain injury patients: a randomized clinical trial. *Annals of General Psychiatry*, *20*(1), 33.

Smits, F. M., Geuze, E., Schutter, D. J., van Honk, J., & Gladwin, T. E. (2022). Effects of tDCS during inhibitory control training on performance and PTSD, aggression and anxiety symptoms: a randomized‐controlled trial in a military sample. *Psychological medicine*, *52*(16), 3964‐3974.

Summerell, E., Xiao, W., Huang, C., Terranova, J., Gilam, G., Riva, P., & Denson, T. F. (2024a). The effects of transcranial direct current stimulation over the ventromedial prefrontal cortex on reactive aggression in intoxicated and sober individuals. *Biological Psychology*, *193*, 108899.

Summerell, E., Bradley, J., Thalody, K., Ford, K., Gilam, G., Riva, P.,… & Summerell, E. (2024b). Transcranial direct current stimulation over the ventrolateral prefrontal cortex improves anger regulation in intoxicated individuals high in trait anger.

Weidler, C., Habel, U., Wallheinke, P., Wagels, L., Hofhansel, L., Ling, S.,… & Clemens, B. (2022). Consequences of prefrontal tDCS on inhibitory control and reactive aggression. *Social cognitive and affective neuroscience*, *17*(1), 120‐130.

Weidler, C., Hofhansel, L., Regenbogen, C., Müller, D., Clemens, B., Montag, C.,… & Habel, U. (2024). The influence of the COMT Val158Met polymorphism on prefrontal TDCS effects on aggression. *Scientific reports, 14*(1), 3437.
